# Falling through the cracks’ – the role of assertive alcohol outreach teams in treating comorbid mental health problems in people with addictions

**DOI:** 10.1192/bjo.2021.736

**Published:** 2021-06-18

**Authors:** E. Naomi Smith, Emily Finch, Colin Drummond

**Affiliations:** South London and Maudsley NHS Foundation Trust

## Abstract

**Aims:**

Input from Assertive Alcohol Outreach Teams (AAOTs) reduces the ‘burden’ on already overstretched community mental health teams (CMHTs).

AAOTs are specialist addictions services. This project focuses on an AAOT based in the London, which engages with people with severe alcohol and illicit substance misuse problems.

Previous research has shown that input from AAOTs reduces hospital admissions. This project examined the impact of AAOT input on reducing the ‘burden’ on CMHTs.

**Method:**

The full caseload of the Southwark-based AAOT was reviewed, including mental health records, general practitioner notes, hospital notes and discharge summaries. We collected data on diagnoses and previous hospital admissions. Patients were assessed to determine whether they met criteria to be open to a CMHT (the presence of complex or serious mental health problems, in addition to addictions).

**Result:**

The caseload was made up of 39 patients, 85% of patients were deemed to meet criteria for being under the care of a CMHT. Only 15% of patients are currently under the care of a CMHT. 87% of patients had at least one comorbid psychiatric diagnosis. 72% of patients had had at least one emergency department or medical hospital admission due to mental health-related problems. 39% had previous admissions to mental health wards. 21% of patients has been admitted under Section of Mental Health Act.

**Conclusion:**

The majority of AAOT patients have severe mental health problems in addition to addictions. The patients are complex and often have a history of disengagement from standard mental health services. Formal diagnosis and treatment of comorbid mental health problems is challenging in the presence of protracted drug and alcohol misuse. AAOT input appears to address a serious ‘gap’ in supporting patients with complex mental health needs who are often ineligible for CMHT input or disengage from CMHT support.

